# Proteomic and Systematic Functional Profiling Unveils Citral Targeting Antibiotic Resistance, Antioxidant Defense, and Biofilm-Associated Two-Component Systems of Acinetobacter baumannii To Encumber Biofilm and Virulence Traits

**DOI:** 10.1128/mSystems.00986-20

**Published:** 2020-11-17

**Authors:** Anthonymuthu Selvaraj, Alaguvel Valliammai, Pandiyan Muthuramalingam, Sivasamy Sethupathy, Ganapathy Ashwinkumar Subramenium, Manikandan Ramesh, Shunmugiah Karutha Pandian

**Affiliations:** a Department of Biotechnology, Alagappa University, Karaikudi, Tamil Nadu, India; b Department of Systems Biology, Science Research Centre, Yonsei University, Seoul, South Korea; c Biofuels Institute, School of the Environment and Safety Engineering, Jiangsu University, Zhenjiang, Jiangsu, China; d College of Medicine, Pennsylvania State University, Hershey, Pennsylvania, USA; California State University, Fresno

**Keywords:** *Acinetobacter baumannii*, citral, biofilm, two-dimensional gel electrophoresis, MALDI-TOF/TOF, omics-based approaches

## Abstract

Acinetobacter baumannii has been reported as a multidrug-resistant bacterium due to biofilms and antimicrobial resistance mechanisms. Hence, novel therapeutic strategies are necessary to overcome A. baumannii infections. This study revealed that citral at 200 μg/ml attenuated A. baumannii biofilms by up to 90% without affecting viability. Furthermore, microscopic analyses and *in vitro* assays confirmed the antibiofilm efficacy of citral. The global effect of citral on A. baumannii was evaluated by proteomic, transcriptional, and *in silico* approaches. Two-dimensional (2D) gel electrophoresis and matrix-assisted laser desorption ionization–time of flight/time of flight (MALDI-TOF/TOF) analyses were used to assess the effect of citral on the A. baumannii cellular proteome. Quantitative real-time PCR (qPCR) analysis was done to validate the proteomic data and identify the differentially expressed A. baumannii genes. Protein-protein interactions, gene enrichment, and comparative gene network analyses were performed to explore the interactions and functional attributes of differentially expressed proteins of A. baumannii. Global omics-based analyses revealed that citral targeted various mechanisms such as biofilm formation, antibiotic resistance, antioxidant defense, iron acquisition, and type II and type IV secretion systems. The results of antioxidant analyses and antibiotic sensitivity, blood survival, lipase, and hemolysis assays validated the proteomic results. Cytotoxicity analysis showed a nontoxic effect of citral on peripheral blood mononuclear cells (PBMCs). Overall, the current study unveiled that citral has multitarget efficacy to inhibit the biofilm formation and virulence of A. baumannii.

**IMPORTANCE** Acinetobacter baumannii is a nosocomial-infection-causing bacterium and also possesses multidrug resistance to a wide range of conventional antibiotics. The biofilm-forming ability of A. baumannii plays a major role in its resistance and persistence. There is an alarming need for novel treatment strategies to control A. baumannii biofilm-associated issues. The present study demonstrated the strong antibiofilm and antivirulence efficacy of citral against A. baumannii. In addition, proteomic analysis revealed the multitarget potential of citral against A. baumannii. Furthermore, citral treatment enhances the susceptibility of A. baumannii to the host innate immune system and reactive oxygen species (ROS). Cytotoxicity analysis revealed the nonfatal effect of citral on human PBMCs. Therefore, citral could be the safest therapeutic compound and can be taken for further clinical evaluation for the treatment of biofilm-associated infections by A. baumannii.

## Abstract (precis)

Acinetobacter baumannii is a nosocomial-infection-causing bacterium and also possesses multidrug resistance to a wide range of conventional antibiotics. The biofilm-forming ability of A. baumannii plays a major role in its resistance and persistence. There is an alarming need for novel treatment strategies to control A. baumannii biofilm-associated issues. The present study demonstrated the strong antibiofilm and antivirulence efficacy of citral against A. baumannii. In addition, proteomic analysis revealed the multitarget potential of citral against A. baumannii. Furthermore, citral treatment enhances the susceptibility of A. baumannii to the host innate immune system and reactive oxygen species (ROS). Cytotoxicity analysis revealed the nonfatal effect of citral on human PBMCs. Therefore, citral could be the safest therapeutic compound and can be taken for further clinical evaluation for the treatment of biofilm-associated infections by A. baumannii.

## INTRODUCTION

Acinetobacter baumannii is a coccobacillus Gram-negative bacterium that causes nosocomial infections such as bacteremia, meningitis, wound infections, urinary tract infections, and pneumonia. It is mostly identified in hospital-associated infections worldwide. Initially, it was referred to as Iraqibacter due to untreatable infection spread by A. baumannii among injured U.S. military soldiers in a civilian hospital in Iraq (1). The Infectious Diseases Society of America categorized A. baumannii as one of the pathogens in the “ESKAPE” group of bacteria. This multidrug-resistant (MDR) bacterium has been noted as a high-priority pathogen by the World Health Organization (WHO) in a report about the list of MDR bacteria for the development of new drugs (2). Several antibiotic resistance mechanisms have been identified in A. baumannii, such as quorum sensing, biofilm formation, efflux pumps, and alteration of the outer membrane. Biofilm formation is one of the most important factors for the persistent survival on various surfaces and the antibiotic resistance of A. baumannii (3).

In a biofilm, bacterial communities are attached to biotic and abiotic surfaces and surrounded by a matrix of extracellular polymeric substances (EPSs), which include polysaccharides, proteins, extracellular DNA, and virulence factors. EPS acts as a barrier to antibiotics, and it provides more survival tolerance against environment stress and the human immune system. Therefore, bacteria in biofilms are much more resistant than planktonic cells. Moreover, antibiotic failure due to altered phenotypes of bacteria in biofilms increases the morbidity and mortality rates in health care units (4, 5). Thus, there is a need for the discovery of alternative strategies to treat biofilm-associated bacterial infections. In A. baumannii, outer membrane proteins, the two-component system (TCS) (*bfmS* and *bfmR*), the chaperone-usher system, quorum sensing, poly-β-(1,6)-*N*-acetylglucosamine (PNAG), phospholipase, the secretion of serine proteases, and motility are various virulence systems associated with biofilm formation (6). Hence, targeting biofilm formation by A. baumannii is an alternative way to control biofilm-mediated infections.

A potential antibiofilm agent will have the ability to inhibit biofilm formation or disperse preformed biofilms, thereby inhibiting virulence factor expression in pathogenic bacteria. In addition, it will not affect bacterial survival, and hence, the chances of resistance development against antibiofilm agents by bacteria will be reduced (7). Conventionally, natural resources have always proven to be a predominant reservoir of therapeutic agents. The exploration of bioactive compounds from natural sources acquires an added advantage since they are expected to be nontoxic (8). Hence, various natural resources have already been explored for their ability to inhibit biofilm-associated virulence factors of clinically important human pathogens, such as Actinidia deliciosa, Syzygium cumini, Macaranga tanarius, and Vetiveria zizanioides extracts against Serratia marcescens, *Candida* spp., A. baumannii, and Staphylococcus aureus, respectively (9–12). Therefore, the identification of antibiofilm agents from natural sources is expected to aid in the development of therapeutic strategies against A. baumannii. Thus, the current study mainly focused on screening natural resources with antibiofilm activity against A. baumannii.

Essential oils from plants are well known to have biological activities. Citral is a mixture of monoterpenoid aldehydes such as neral and geranial. It has been known for its pleasant odor, and it is abundantly found in Cymbopogon citratus, lemon myrtle, Citrus limon, and Aloysia triphylla. It has been used for its wide range of biological properties, such as its antimicrobial, antifungal, anti-inflammatory, antimutagenic, antioxidant, and anticancer activities (13–15). Previous studies reported the antibiofilm activity of citral against Staphylococcus aureus, Listeria monocytogenes, Vibrio parahaemolyticus, and Candida tropicalis (16–19). With this backdrop, the current study aimed at evaluating the antibiofilm potential of citral against A. baumannii and mainly focused on a proteomics-based approach to decipher the molecular mechanism underlying the antibiofilm activity of citral.

## RESULTS

### Growth and biofilm formation of A. baumannii upon phytocompound treatment.

**FIG 1:**
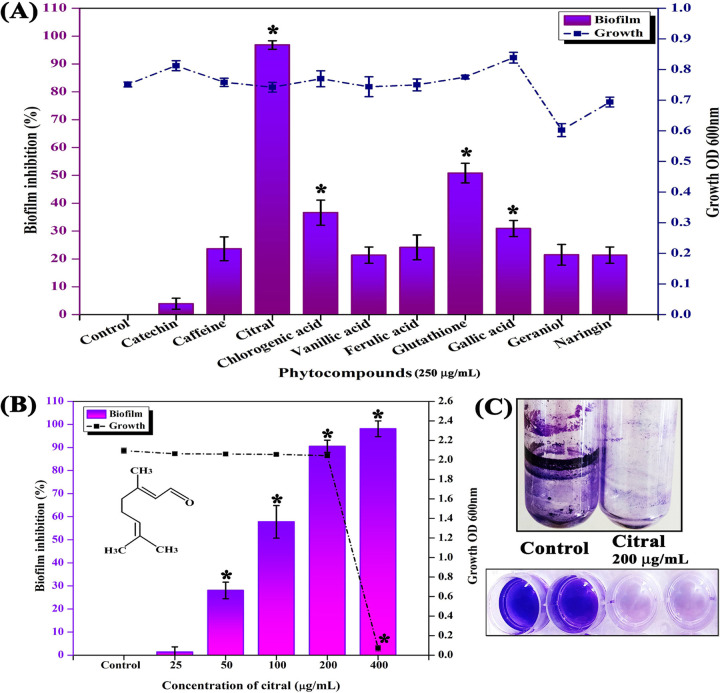
Screening of antibiofilm activities of phytocompounds and identification of the MIC and MBIC of citral. (A) Effect of phytocompounds (250 μg/ml) on growth and biofilm formation of A. baumannii. (B) Dose-dependent effect of citral on growth and biofilm formation of A. baumannii. (C) Citral (200 mg/ml) inhibiting biofilm formation of A. baumannii on polystyrene and glass surfaces. Error bars and asterisks indicate the SD and statistical significance (*P ≤ *0.05), respectively.

The antibiofilm potentials of 10 phytocompounds were tested against A. baumannii at 250 μg/ml. Out of 10 phytochemicals, citral at 250 μg/ml showed strong antibiofilm activity (97%) without disturbing the growth of A. baumannii. The rest of the phytocompounds at 250 μg/ml showed insignificant changes in the cell density as well as biofilm formation of A. baumannii (Fig. 1A).

### Effect of the MIC and MBIC of citral.

The MIC and minimal biofilm-inhibitory concentration (MBIC) of citral were assessed at increasing concentrations (25, 50, 100, 200, and 400 μg/ml) against A. baumannii. Citral at 200 μg/ml exhibited 90% antibiofilm activity, above which antibacterial activity was observed. Thus, 200 μg/ml was considered the MBIC. At a 400-μg/ml concentration, citral showed strong antibacterial activity against A. baumannii, and hence, the same was fixed as the MIC (Fig. 1B).

### Inhibitory effect of citral on A. baumannii biofilm formation on various surfaces.

The effect of citral on the biofilm formation of A. baumannii on glass (test tubes) and polystyrene (24-well plate) surfaces was evaluated. The results showed that citral at 200 μg/ml effectively inhibited the biofilm formation of A. baumannii on glass as well as 24-well polystyrene surfaces (Fig. 1C).

### Nonantibacterial effect of citral on A. baumannii.

**FIG 2:**
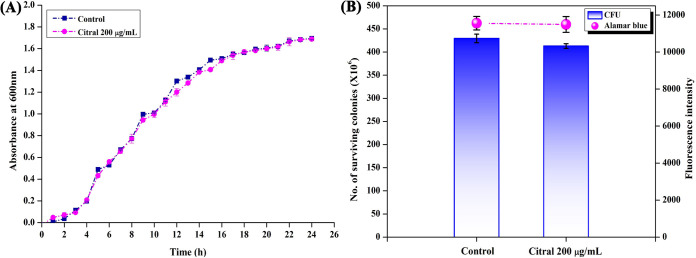
Effect of citral on A. baumannii growth and viability. (A) Influence of the MBIC of citral on the growth curve of A. baumannii. (B) Nonfatal effect of citral on the metabolic viability of A. baumannii assessed by Alamar Blue and CFU analyses. Error bars indicate the SD.

The growth curves of A. baumannii in the absence and presence of citral (200 μg/ml) were obtained at 600 nm for every hour up to 24 h. An insignificant change in the growth curve patterns of control and treated samples was observed (Fig. 2A). The effect of citral on the cell viability of A. baumannii was analyzed by an Alamar Blue assay to compare control and citral-treated cells. The result revealed insignificant differences between the control and treated samples in terms of cell viability (Fig. 2B). Also, the numbers of viable cells in control and treated samples were assessed, and Fig. 2B shows insignificant changes in cell counts in control (4.3 × 10^8^ cells) and citral-treated (4.1 × 10^8^ cells) cultures. Thus, the growth curve, CFU, and Alamar Blue assays confirmed the insignificant effect of citral on the growth and viability of A. baumannii.

### Microscopic analyses of A. baumannii biofilm formation.

**FIG 3:**
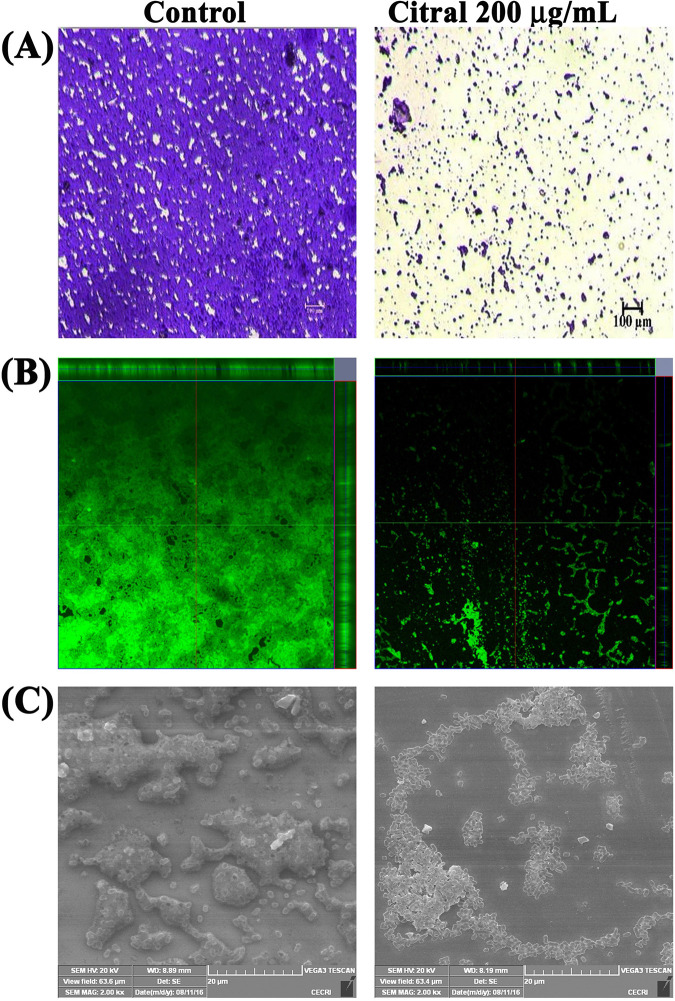
Microscopic analyses of the antibiofilm activity of citral. (A and B) Light (A) and CLSM (B) micrographs exhibiting the antibiofilm efficacy of citral on A. baumannii. (C) SEM analysis displaying the inhibitory effect of citral on multilayered biofilm formation by A. baumannii.

Microscopic analyses were performed to evaluate the biofilm architecture of A. baumannii in the absence and presence of citral at a 200-μg/ml concentration. The light microscopy results clearly showed a reduction in the covered surface area of the biofilm on the glass slide in the case of citral treatment compared to the control sample (Fig. 3A). The biofilm thickness of control and citral-treated samples was analyzed and visualized by confocal laser scanning microscopy (CLSM). Multilayered biofilm formation was observed in the CLSM image of the control sample, whereas the citral-treated sample showed a very thin and dispersed biofilm (Fig. 3B). Furthermore, scanning electron microscopy (SEM) analysis was performed to assess the surface morphology of the A. baumannii biofilm. The SEM image of the control sample displayed highly aggregated cells with a thick layer of EPS. In contrast, the SEM image of the citral-treated sample depicted a reduction in EPS production and a disintegrated biofilm (Fig. 3C). Altogether, the microscopic analyses further validated the antibiofilm efficacy of citral against A. baumannii.

### Inhibitory effect of citral on EPS and CSH of A. baumannii.

**FIG 4:**
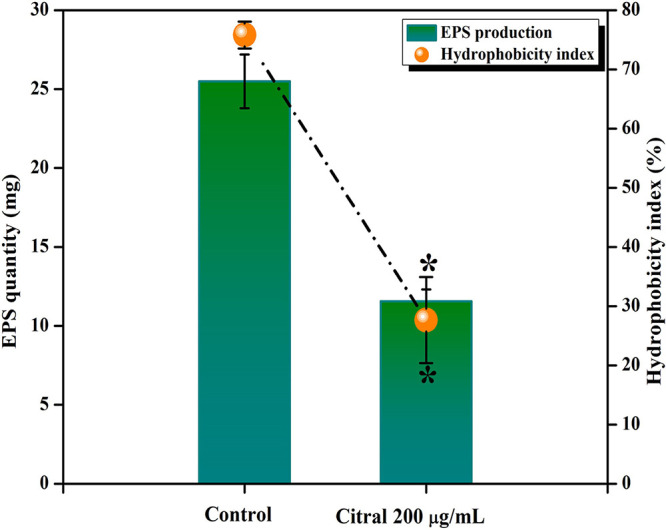
Influence of citral on CSH and EPS production in A. baumannii. Citral treatment significantly inhibited the CSH and EPS production in A. baumannii. Error bars and asterisks indicate the SD and statistical significance (*P ≤ *0.05), respectively.

The effect of citral on the EPS production of A. baumannii was assessed, and EPS extracted from the citral (200-μg/ml)-treated sample showed a maximum of a 44% reduction (11.5 ± 0.75 mg) compared to the control sample (25.5 ± 1.2 mg) (Fig. 4). Furthermore, a microbial adherence to hydrocarbon (MATH) assay was performed to determine the inhibitory eﬀect of citral on the cell surface hydrophobicity (CSH) of A. baumannii. The results revealed that the addition of citral at 200 μg/ml significantly reduced the CSH of A. baumannii (27%) compared to the CSH of the control sample (76%) (Fig. 4).

### Identification of differentially expressed proteins of A. baumannii.

**FIG 5:**
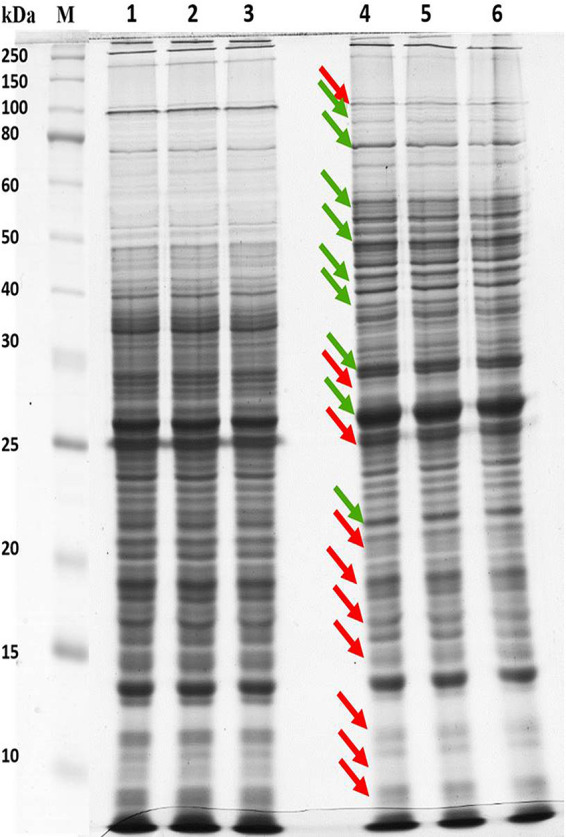
One-dimensional gel electrophoresis and qPCR analysis. SDS-PAGE gels show the cellular protein profiles of control and citral-treated samples. Lane M corresponds to the marker protein. Lanes 1 to 3 and lanes 4 to 6 represent the control and citral-treated protein samples, respectively. Green and red arrows indicate the up- and downregulation of proteins upon citral treatment, respectively.

**FIG 6:**
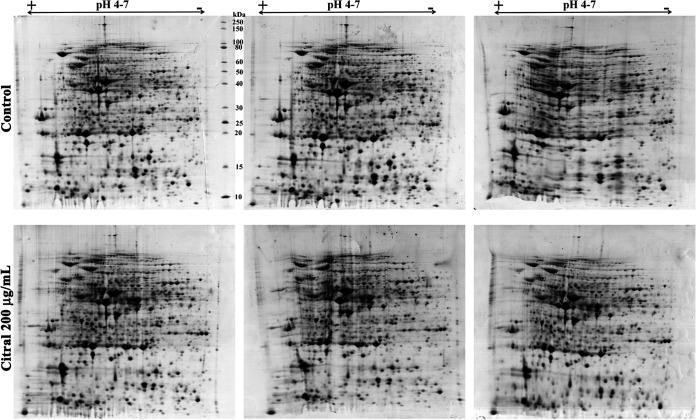
Effect of citral on the cellular proteome profile of *A. baumannii* analyzed by 2DGE in biological triplicates.

**FIG 7:**
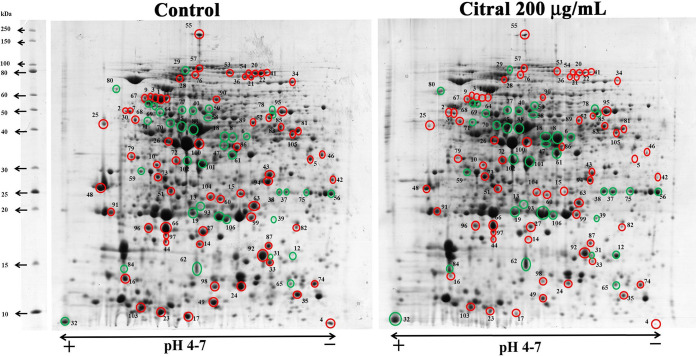
Comparative cellular proteomic profiling by 2DGE. Shown are cellular proteome profiles of control and citral-treated samples after isoelectric focusing (18-cm IPG strip [pH 4 to 7]) and separation by 12% SDS-PAGE. After staining, ImageMaster 2D Platinum software was used to identify the differentially expressed protein spots. Up- and downregulated spots are marked with green and red circles, respectively.

Initially, sodium dodecyl sulfate-polyacrylamide gel electrophoresis (SDS-PAGE) analysis was carried out to analyze the intracellular protein samples isolated from A. baumannii grown in the absence and presence of citral (200 μg/ml). The differentially expressed bands were observed in citral-treated samples. The up- and downregulated bands are marked by green and red arrows, respectively, in Fig. 5. To separate proteins with high resolution, two-dimensional gel electrophoresis (2DGE) of the intracellular proteomes of control and citral-treated samples was performed in biological triplicates (Fig. 6). Next, protein spots were analyzed using ImageMaster 2D Platinum software, and 892 protein spots were identified to be differentially regulated. Among 892 protein spots, 70 downregulated (red circles) and 36 upregulated (green circles) (≥1.5-fold change; *P < *0.05) spots were selected for further analysis (Fig. 7). The differentially expressed proteins were subjected to trypsin digestion, analyzed by matrix-assisted laser desorption ionization–time of flight/time of flight (MALDI-TOF/TOF) spectrometry, and identified using the MS-Fit online database. Details of the differentially expressed proteins are given in Tables 1 and 2.

**TABLE 1:** Upregulated proteins of A. baumannii treated with citral

Serial no.	Spot no.	Fold change	*P* value by ANOVA	MOWSE score^*a*^	Coverage (%)	No. of peptides matched	UniProt ID	Description	Gene
1	6	9.80019	0.0072063	429,407	34	10	D0C713	Chlorophyll synthesis pathway protein BchC	F898_02608
2	8	8.71292	0.00691163	80,765	22.9	10	D0C713	Chlorophyll synthesis pathway protein BchC	F898_02609
3	12	5.51743	0.00371512	212	33.3	4	D0C711	Cupin domain protein	yhhW_3
4	18	4.34739	0.005373	2,941	9.4	6	D0C713	Chlorophyll synthesis pathway protein	F962_01825
5	19	4.29623	1.80E−04	3.47E+09	35.4	18	D0C712	Carboxymuconolactone decarboxylase	*pcaC*
6	29	2.79884	0.00497461	5,452	7	12	D0C9Q7	FtsK/SpoIIIE family protein	J663_1111
7	31	2.754	0.0289272	757	28	4	D0C711	Cupin domain protein	yhhW_2
8	32	2.68892	0.0346535	1,723	11	7	D0C923	Uncharacterized protein	HMPREF0010_01581
9	37	2.45658	0.0277662	16,963	25.3	9	D0CBH7	Electron transfer flavoprotein subunit beta	*etfB*
10	38	2.4521	0.0110412	8,938	25	14	N9JYT0	Xanthine phosphoribosyltransferase	*xpt*
11	39	2.45149	0.00325003	6.23E+06	41	15	D0CBH7	Electron transfer flavoprotein subunit beta	*etfB*
12	40	2.43191	0.0963101	2.17E+07	30.8	23	D0CEK6	ATP synthase subunit alpha	*atpA*
13	45	2.18798	0.0031792	1.15E+06	24	11	D0CG85	Elongation factor Tu (fragment)	*tuf*
14	47	2.15711	0.0244955	1.48E+08	42.9	18	D0CAC5	Ketol acid reductoisomerase (NADP^+^)	*ilvC*
15	50	2.10401	0.0247724	1.62E+07	27.9	17	D0CE42	1-Pyrroline dehydrogenase	*putA*
16	56	2.02353	0.00981648	812,305	37	13	D0CBH7	Electron transfer flavoprotein subunit beta	*etfB*
17	58	2.00514	0.00416032	181,873	22.8	15	D0CC64	Adenosylhomocysteinase	*ahcY*
18	59	1.97109	0.0305157	28,488	24	12	D0CBH8	Electron transfer flavoprotein subunit alpha	*etfA*
19	61	1.94181	0.0030139	8,564	6	6	D0CDT6	Succinate-CoA ligase (ADP-forming) subunit alpha	*sucD*
20	62	1.93165	0.00672927	4,654	28	6	D0C6C9	Biotin carboxyl carrier protein of acetyl-CoA carboxylase	*accB*
21	64	1.90147	0.030785	1.57E+13	30	21	D0CEK4	ATP synthase subunit beta	*atpD*
22	65	1.88344	0.024857	14,151	46	6	D0C6D0	3-Dehydroquinate dehydratase	*aroQ*
23	68	1.86333	0.0681003	6,537	14	9	N9JSL7	Uncharacterized protein	Unknown
24	70	1.84877	0.03694	3.45E+07	27	14	N9JNN0	Elongation factor Tu	*tuf*
25	71	1.84746	0.0228597	3.69E+07	48.6	20	D0CFL0	Cell division protein FtsZ	*ftsZ*
26	75	1.79722	0.002872	2.54E+09	55.5	19	D0C612	Oxidoreductase, short-chain dehydrogenase/reductase family protein	EA682_19600
27	77	1.78907	0.0224894	533,427	25.9	19	N9LHG7	ATP synthase subunit alpha	*atpA*
28	78	1.76984	0.0275672	4,384	20.8	12	D0CDT4	Dihydrolipoyl dehydrogenase	*lpdA*
29	80	1.7645	0.0396424	6.58E+07	23	18	D0CAX2	Transcription termination/antitermination protein NusA	*nusA*
30	88	1.69679	0.014505	1.99E+06	30.3	12	D0CG85	Elongation factor Tu (fragment)	*tuf*
31	84	1.71455	0.038369	1,606	21	8	N9LCE6	Uncharacterized protein	Unknown
32	85	1.7089	0.086881	4.22E+06	32	16	N9LF65	Uncharacterized protein	Unknown
33	93	1.64787	0.0236251	2,946	22	9	D0C775	Uncharacterized protein	HMPREF0010_00605
34	101	1.56314	0.040766	13,868	8.4	8	D0CB50	Glutamyl-Q tRNA(Asp) synthetase	*gluQ*
35	102	1.55785	0.00506707	6,223	14.7	7	D0CB97	Glycosyl hydrolase family 25	J506_1918
36	106	1.54278	0.0629351	49,184	26.1	10	D0CAG8	Uncharacterized protein	HMPREF0010_0174

a MOWSE, MOlecular Weight SEarch.

**TABLE 2:** Downregulated proteins of A. baumannii treated with citral^*a*^

Serial no.	Spot no.	Fold change	*P* value by ANOVA	MOWSE score	Coverage (%)	No. of peptides matched	UniProt ID	Description	Gene(s)
1	1	21.2936	0.0172848	4.75E+07	27.9	23	D0CBD9	Chaperonin	*groL*
2	2	16.8399	4.82E−04	1.21E+07	18	28	D0CBD9	Chaperonin	*groL*
3	3	15.865	0.028111	5.77E+06	19.7	19	D0CBD9	Chaperonin	*groL*
4	4	10.9888	0.00202535	1,880	30.6	5	D0C944	Uncharacterized protein	HMPREF0010_01602
5	5	10.0257	0.001597	2,182	22	6	D0CCN2	Putative rRNA large-subunit methyltransferase	*rlmH*
6	7	8.73838	0.00939942	1.69E+06	17	21	D0CBD9	Chaperonin	*groL*
7	9	8.41899	0.00134163	121,830	20.2	19	D0CBD9	Chaperonin	*groL*
8	10	8.26801	0.0170282	45,779	20.2	17	D0C5P3	Type II secretion system protein L	A7M90_04465
9	11	7.68321	0.039143	1.69E+06	68	17	D0CEH1	Chaperonin	*clpB*
10	14	5.42105	0.00759725	28,386	62.7	10	D0C6U1	Ring-hydroxylating beta subunit	EA682_14025
11	13	5.51553	0.0422398	2,409	12	5	D0C8D9	3-Oxoadipate CoA-transferase subunit B	*pcaJ*
12	15	5.3114	0.0258016	3,449	30.8	6	D0CC56	Superoxide dismutase	*sodB*
13	16	4.68706	0.00457015	259,521	31.3	9	D0CDE9	Glyoxalase family protein	HMPREF0010_02779
14	17	4.51329	0.00236441	57,717	26.5	8	D0C694	Universal stress protein	*uspA*
15	20	3.90633	0.00494997	2.42E+11	21	25	D0C8B2	Catalase	*katE*
16	21	3.86299	0.0155406	2.92E+07	19.2	17	D0CBU4	Isocitrate dehydrogenase	*idh*
17	22	3.78332	0.0043499	3,267	10	8	D0CCF0	AAA_13 domain-containing protein	HMPREF0010_02430
18	23	3.69536	0.00588856	15,744	26.5	7	D0C694	Universal stress protein	*uspA*
19	24	3.60009	0.0389191	11,124	6.5	10	D0C8Q7	Urea carboxylase	*uca*
20	25	3.07919	0.0108388	130,244	32	12	D0CBN6	Uncharacterized protein	HMPREF0010_02166
21	26	2.96261	0.0136496	5.44E+06	20.5	14	D0CDF2	Outer membrane protein OmpA	*ompA*
22	27	2.85325	2.62E−04	15,876	25	9	D0CB44	TetR family transcriptional regulator	A7M90_07770
23	28	2.81951	0.0154604	3,291	12.5	11	D0C7C2	TonB-dependent siderophore receptor	B4R90_17225
24	30	2.75473	0.00636684	1.50E+06	15	12	D0CBD9	60-kDa chaperonin	*groL*
25	33	2.68762	0.0248783	1,058	5.2	13	D0CCP2	Glutamate synthase	*gltA*
26	34	2.68642	0.0391656	1.34E+10	13.3	23	N9L1T6	Phenylacetic acid degradation protein PaaN	*paaZ*
27	35	2.51316	0.0045256	726,037	72.9	15	N9JSL3	Phenylacetic acid degradation protein PaaB	*paaB*
28	36	2.51043	0.0278942	7,781	11	12	D0CBU4	Isocitrate dehydrogenase, NADP dependent	*icd*
29	41	2.43079	0.0209266	1.92E+10	22.2	24	D0C8B2	Catalase	*katG*
30	42	2.28891	0.020157	2,448	25.9	8	D0CC77	AraC-like ligand binding domain protein	AOLE_05420
31	43	2.2692	0.00503751	1.13E+09	37	17	N9JSK9	Uncharacterized protein	
32	44	2.20261	0.02930955	9.76E+06	41.2	13	D0CFW7	Alkyl hydroperoxide reductase	*ahpC*
33	46	2.16065	0.00773051	55,904	20.3	11	D0C6S7	LysR family transcriptional regulator	*cynR_3*
34	48	2.11846	0.0177177	3.20E+07	40	17	D0CF50	Uncharacterized protein	HMPREF0010_03516
35	49	2.11518	0.0503401	21,781	43.5	9	D0CB31	H-NS histone family protein	HMPREF0010_01961
36	51	2.09808	0.0265194	5.89E+06	26.3	16	D0CDD0	Antioxidant, AhpC/TSA family	*ahpC*
37	52	2.07166	0.00764134	1,072	14.2	9	D0C6F4	Fumarate hydratase class II	*fumC*
38	53	2.06832	0.0252401	1.23E+09	16.2	21	D0C7P8	Malate synthase G	*glcB*
39	54	2.05527	0.02606	3.01E+10	21	24	D0C8B2	Catalase	*katA*
40	55	2.05413	0.0880032	8,311	13	16	D0CB16	DNA-directed RNA polymerase subunit beta′	*rpoC*
41	57	2.00582	0.0128014	2.67E+08	19.1	17	D0CEH1	Chaperone protein ClpB	*clpB*
42	60	1.96427	0.0322003	824	24.4	4	D0C902	Adenylate kinase	*adk*
43	63	1.92323	5.69E−05	938,316	35.4	10	D0CDK4	Intracellular protease, PfpI family	*yraA*
44	66	1.86778	0.00254709	7,587	39	8	D0C9K9	LysM domain protein	J529_2291, *plc*, *plcD*
45	67	1.86611	0.0057751	7.72E+08	17	14	D0CDA7	Acyl-CoA dehydrogenase, C-terminal domain protein	*acdB2*
46	69	1.86333	0.0381003	3.47E+07	20.7	9	N9L061	Alkyl hydroperoxide reductase	*ahpC*
47	72	1.82905	0.0182108	5.76E+09	32.3	18	D0CD53	Malate dehydrogenase	*mdh*
48	73	1.82434	0.0183562	2.59E+06	30.3	13	D0C9D6	RstA transcriptional regulatory protein	*rstA*-*bfmR*
49	74	1.82132	0.0735539	6.21E+06	37	15	N9JXR1	Universal stress protein	*uspA*
50	76	1.79174	7.36E−04	252,631	20	15	D0CEH1	Chaperone protein ClpB	*clpB*
51	79	1.76828	0.00421035	168,184	35.3	16	D0C6V4	Catechol 1,2-dioxygenase	*catA*
52	81	1.74667	0.0141531	2.14E+11	44	23	D0C8J7	3-Oxoadipyl-CoA thiolase	*pcaF*
53	82	1.74038	0.0429863	1.19E+07	17	35	D0CF81	Single-stranded DNA binding protein	*ssb*
54	83	1.73308	0.0323611	55,140	17.3	13	D0CCU3	Glutamate dehydrogenase	*gdhA*
55	86	1.70228	0.032938	104,034	23.8	12	D0C7T3	Fructose-bisphosphate aldolase	*fba*
56	87	1.69884	0.035837	8.23E+06	53.7	14	D0CCS3	Hemerythrin HHE cation binding domain protein	HMPREF0010_02553
57	89	1.67691	0.0277549	2.07E+09	33	19	N9JU68	Chaperone SurA	*surA*
58	90	1.67489	0.0255439	4.28E+07	24	19	D0C8Y6	Isocitrate lyase	*aceA*
59	91	1.67054	0.2635	4.28E+07	24	19	D0C8Y6	Isocitrate lyase	*aceA*
60	92	1.66276	0.0106452	270,816	27	12	D0CBN6	Uncharacterized protein	HMPREF0010_02166
61	94	1.62852	0.038033	3,987	14.5	7	D0CAV6	Uncharacterized protein	HMPREF0010_00605
62	95	1.61363	0.0560761	10,884	21.5	9	D0C762	Response regulator receiver domain protein	*adeRS*, *adeG*, *adeH*
63	96	1.6036	0.0276446	3,663	14.4	15	D0CDR1	Siroheme synthase	*cobA*
64	97	1.59878	0.014937	6,099	34	8	D0C9J0	Bacterioferritin	*bfr*
65	98	1.59438	0.0315863	891	23.1	4	D0C694	Universal stress protein	*uspA*
66	99	1.59331	0.0210223	2,012	16.2	5	D0C966	Uncharacterized protein	HMPREF0010_01624
67	100	1.57856	0.034285	2,645	19.8	6	D0CDV3	Uncharacterized protein	HMPREF0010_02933
68	103	1.54355	0.0246468	1,309	47.9	7	D0C8A0	Acetyltransferase, GNAT family	*gcuP*
69	104	1.53001	0.036002	2,056	14.4	11	D0C7T9	TetR family transcriptional regulator	*kstR2_3*
70	105	1.51411	0.0104371	1,743	4.6	8	D0CF95	Efflux transporter, RND family, MFP subunit	*adeF*

a MOWSE, MOlecular Weight SEarch; TSA, thiol-specific antioxidant; GNAT, Gcn5-related *N*-acetyltransferases; RND, resistance-nodulation-division; MFP, membrane fusion protein.

### PPI and functional enrichment analyses.

**FIG 8:**
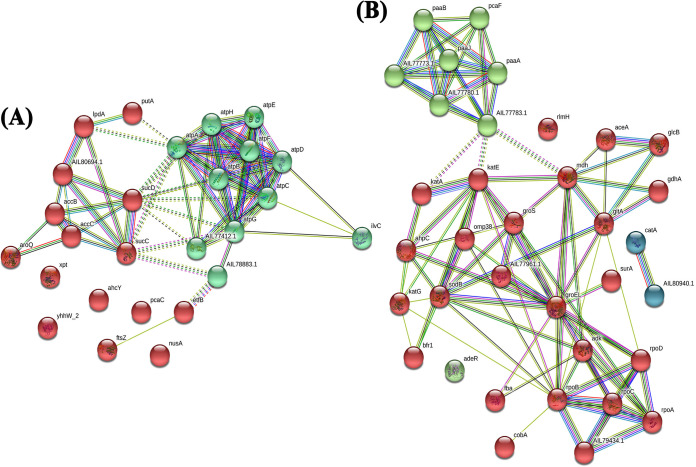
Molecular interaction networks of differentially expressed proteins. The cluster analysis of upregulated (A) and downregulated (B) proteins imputed by STRING v11.0 shows tightly connected functional modules. The identified clusters are in red, teal, green, and blue. The dotted and solid lines represent an interaction/connection within the same and another cluster, respectively. Colored lines and dots indicate various types of interaction evidence (cyan, from curated databases; green, gene neighborhood; blue, gene cooccurrence; pink, experimentally determined; black, coexpression; light blue, protein homology). Enlarged protein nodes that contain a ribbonlike structure represent the availability of protein three-dimensional (3D) structure information.

The molecular interactions of differentially regulated (up- and downregulated) proteins of A. baumannii were predicted using STRING v11.0. The molecular signals of upregulated proteins had 26 nodes and 66 edges (Fig. 8A). The molecular cross talk of these proteins had a nodal degree of ∼5.08 in neighborhood seed proteins. Additionally, interactions of downregulated proteins had 94 edges and 35 nodes, and the nodal degree was ∼5.37 in closely related seed proteins (Fig. 8B). The cross talk of these differentially expressed proteins was analyzed with an enrichment *P* value score of <0.01.

**FIG 9:**
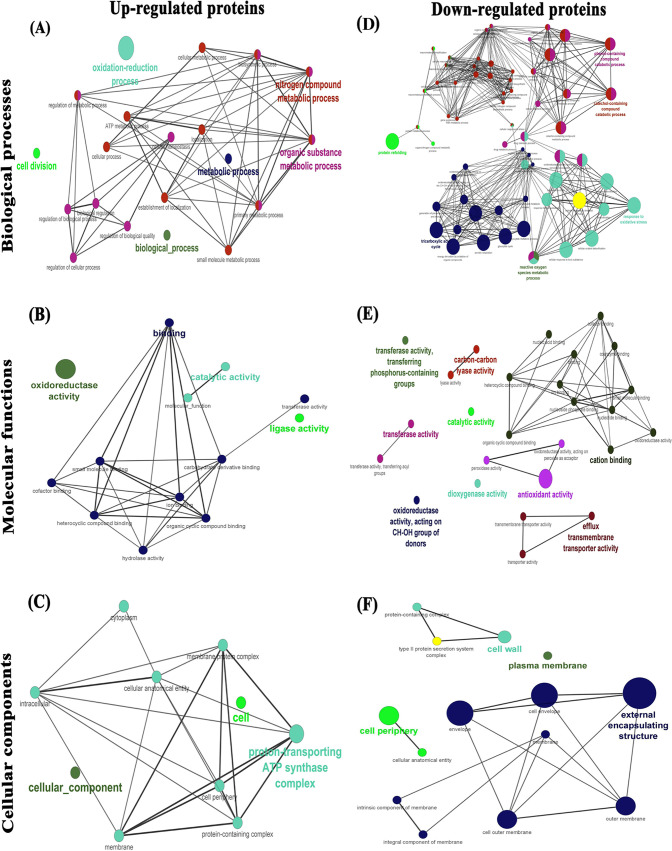
GO functional enrichment analysis of differentially expressed proteins. GO enrichment analysis and visualization of the proteome of A. baumannii (upregulated [A to C] and downregulated [D to F]) were performed using the ClueGO v2.5.7/CluePedia v1.5.7 plug-in for Cytoscape software. The numbers of genes falling in biological processes (A and D), molecular functions (B and E), and cellular components (C and F) are directly proportional to the node size. The node colors correspond to each GO category of differentially expressed proteins according to the significance level of GO terms.

Protein-protein interaction (PPI) networks of differentially regulated proteins and their functional gene enrichments were imputed using ClueGO/CluePedia plug-in of Cytoscape software, and the results revealed that these proteins are significantly involved in diverse biological processes, molecular functions, and cellular components (Fig. 9A to G).

### Effect of citral on biofilm-associated genes of A. baumannii.

**FIG 10:**
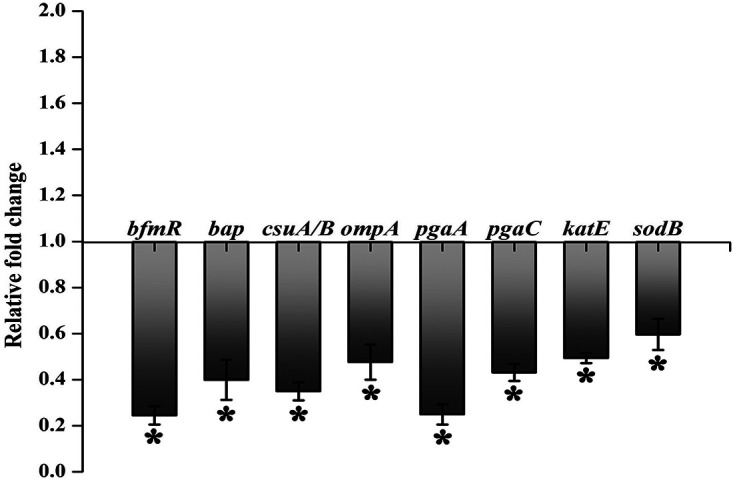
Transcriptional analysis. qPCR analysis reveals the negative regulation of the expression of genes associated with biofilm formation and virulence of A. baumannii under the influence of citral. Error bars and asterisks indicate the SD and statistical significance (*P ≤ *0.05), respectively.

In order to validate the proteomic data and also to assess the effect of citral on biofilm-associated genes of A. baumannii, quantitative real-time PCR (qPCR) analysis was performed, and the results revealed the significant downregulation of the expression of *bfmR*, *bap*, *csuA-csuB*, *ompA*, *pgaA*, *pgaC*, *katE*, and *sodB* (Fig. 10).

### C-G-N analysis.

**FIG 11:**
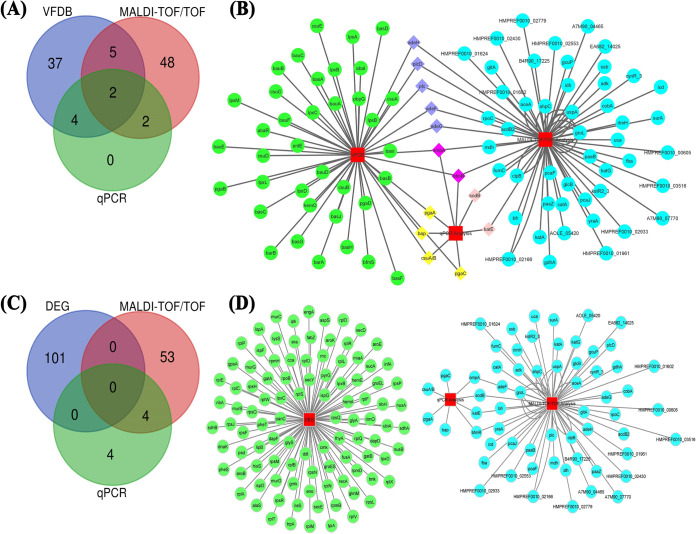
Venn and comparative gene network (C-G-N) analyses. The Venn diagram and network indicate the combinatorial analysis of proteins downregulated by citral identified using MALDI-TOF/TOF and qPCR with the VFDB (A and B) and DEG (C and D).

The Venn intersections and comparative gene network (C-G-N) results shown in Fig. 11A and B consist of 48 virulence factors, 57 unique downregulated protein-coding genes, and 8 downregulated transcriptional genes. Furthermore, the results represent values from 3 possible combinatorial analyses, Virulence Factor Database (VFDB), MALDI-TOF/TOF, and qPCR analyses. Two genes, namely, *ompA* and *bfmR*, were found in VFDB, MALDI-TOF/TOF, and qPCR intersections. VFDB and MALDI-TOF/TOF intersections displayed 5 genes, *adeH*, *adeF*, *adeG*, *plcD*, and *plc*. VFDB and qPCR intersections had 4 genes, *bap*, *csuA*-*csuB*, *pgaC*, and *pgaA*. Two genes, namely, *sodB* and *katE*, were present in MALDI-TOF/TOF and qPCR intersections. Also, 37 genes in VFDB, 48 downregulated genes in MALDI-TOF/TOF, and no unique genes in qPCR analyses were observed without any interaction in systematic network interlinking and Venn intersections.

Among 458 genes in the Database of Essential Genes (DEG), 101 genes that have official gene symbols and known functions were used for Venn and network analyses. No interactions were found between the DEG (101 genes) and all downregulated genes (57 by MALDI-TOF/TOF and 8 by qPCR) (Fig. 11C and D). Furthermore, cross talk was observed only between the MALDI-TOF/TOF and qPCR analyses.

### Influence of citral on the antibiotic sensitivity of A. baumannii.

**FIG 12:**
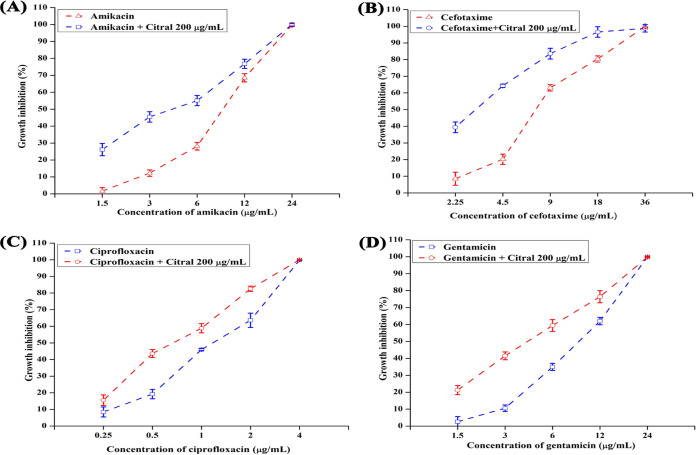
Effect of citral on the susceptibility of A. baumannii to antibiotics. Citral treatment enhanced the sensitivity of A. baumannii to amikacin (A), cefotaxime (B), ciprofloxacin (C), and gentamicin (D). Error bars and asterisks indicate the SD and statistical significance (*P ≤ *0.05), respectively.

The ability of citral (200 μg/ml) to improve the efficacy of antibiotics was tested by a broth microdilution assay. The addition of citral to culture media increased the susceptibility of A. baumannii to various antibiotics such as amikacin, cefotaxime, ciprofloxacin, and gentamicin (Fig. 12).

### Native PAGE analysis of catalase and SOD production.

**FIG 13:**
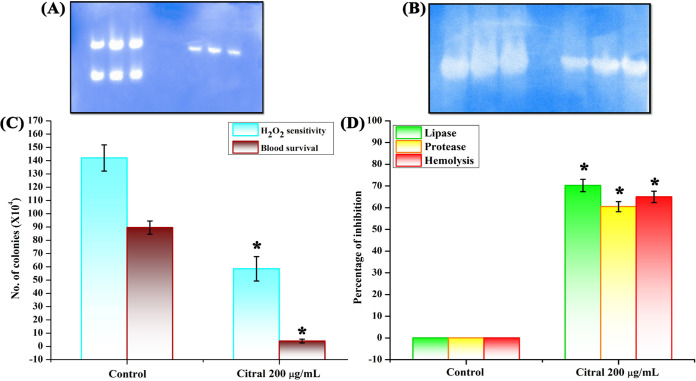
Validation of proteomic data by virulence assays. (A and B) Native PAGE analysis revealing the inhibitory effect of citral treatment on catalase (A) and SOD (B) activities in A. baumannii. (C) Influence of citral on the susceptibility of A. baumannii to H_2_O_2_ and human blood. (D) Inhibitory efficacy of citral on lipase, protease, and hemolysin production. Error bars and asterisks indicate the SD and statistical significance (*P ≤ *0.05), respectively.

Native PAGE was performed to assess the effect of citral on catalase and superoxide dismutase (SOD) production in A. baumannii. The band intensity was reduced for catalase (Fig. 13A) and SOD (Fig. 13B) in citral-treated samples compared to the control samples.

### Impact of citral on the susceptibility of A. baumannii to H_2_O_2_ and healthy human blood.

The sensitivity of A. baumannii to reactive oxygen species (ROS) was determined by an H_2_O_2_ sensitivity assay. The results showed that citral-treated cells were highly sensitive to H_2_O_2_ compared to control cells. Compared to 1.4 × 10^6^ cells that survived in the control sample, 5.8 × 10^5^ viable cells were noticed in the citral-treated sample (Fig. 13C). In addition, the susceptibilities of A. baumannii to healthy human blood were compared between control and citral-treated cells using a blood survival assay. The results showed that killing was abundant upon citral (200 μg/ml) treatment (4 × 10^4^ cells) compared to the control sample (8.9 × 10^5^ cells) (Fig. 13C). Altogether, these results revealed that the resistance of A. baumannii to ROS and human blood was reduced upon treatment with citral.

### Inhibitory effect of citral on lipase, protease, and hemolysis of A. baumannii.

Bacteria produce extracellular lipase as a virulence factor. In this study, the effect of citral on lipase production by A. baumannii was investigated. Citral reduced lipase production by 70% compared to control cells (Fig. 13D). In addition, citral treatment inhibited the proteolytic activity of A. baumannii up to 60% (Fig. 13D). Also, the results of the hemolysis assay revealed that citral treatment affects the hemolysis activity of A. baumannii by 65% compared to the control sample (Fig. 13D).

### Effect of citral on the motility of A. baumannii.

**FIG 14:**
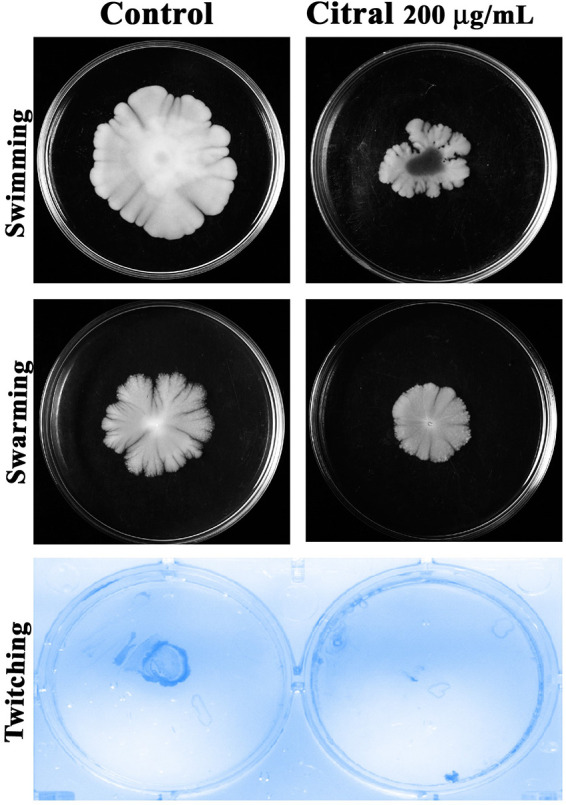
Effect of citral on different types of motility of A. baumannii. Shown are swimming, swarming, and twitching motility of A. baumannii upon citral treatment.

The influence of citral (200 μg/ml) on the motility of A. baumannii was evaluated. Swimming motility was reduced (25 mm) in citral-treated cells compared to control cells (40 mm). In addition, a reduction of swarming motility was observed in treated cells (20 mm) compared to control cells (35 mm). Complete inhibition of twitching motility was observed in treated cells, whereas high twitching motility was observed in control cells (Fig. 14).

### Nonfatal effect of citral on human PBMCs.

**FIG 15:**
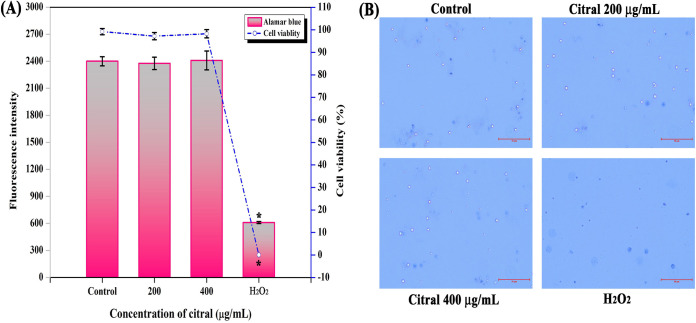
Cytotoxicity analysis of citral. Alamar Blue (A) and trypan blue (B) assays unveil the nonfatal effect of citral on human PBMCs. Error bars and asterisks indicate the SD and statistical significance (*P ≤ *0.05), respectively.

Alamar Blue- and trypan blue-based assays were performed to examine the cytotoxic effect of citral (200 and 400 μg/ml) on human peripheral blood mononuclear cells (PBMCs). The results showed that citral did not cause any harmful effect on the viability of PBMCs, whereas 1 mM H_2_O_2_ completely affected the viability of PBMCs (Fig. 15).

## DISCUSSION

Biofilm-associated infections by A. baumannii are very difficult to cure by conventional antibiotic treatments, and hence, the focus on antibiofilm therapy-based research has been increasing in recent times (20). The present study revealed for the first time that citral inhibits the biofilm formation and virulence factor production of A. baumannii without causing any harmful effect on viability. In addition, this study explored the mode of action underlying the antibiofilm activity of citral. Initially, 10 phytocompounds (250 μg/ml) were screened for antibiofilm potential against A. baumannii. Of the 10 phytocompounds tested, citral most effectively inhibited (97%) the biofilm formation of A. baumannii without affecting growth (Fig. 1A). Citral is approved by the Food and Drug Administration (FDA) for use as a flavoring agent in food industries and is also known to have various biological activities (21). From the present study, citral was found to inhibit A. baumannii biofilms in a concentration-dependent manner, and the MBIC (200 μg/ml) of citral showed a maximum of 90% biofilm inhibition (Fig. 1B). Furthermore, the nonbactericidal effect of citral on A. baumannii was validated by growth curve, CFU, and Alamar Blue assays (Fig. 2), which confirmed that the antibiofilm activity of citral was not through bactericidal activity.

Furthermore, reductions in biofilm-covered areas and biofilm thickness were observed in light microscopy and CLSM images of citral-treated samples, respectively, which further validated the antibiofilm potential of citral against A. baumannii. Furthermore, SEM analysis (Fig. 3) revealed a reduction in biofilm formation with dispersed cells and a low abundance of EPS in citral-treated samples, indicating that citral may have an effect on EPS production. The dry weight of the biofilm matrix contains about 90% EPS, which plays a vital role in biofilm stability and supports bacterial survival on various surfaces. Also, EPS blocks the penetration of antibiotics and protects bacteria from the host immune system (22). Therefore, the influence of citral on EPS production was assessed, and the results depicted a reduction in EPS production by citral treatment (Fig. 4). EPS significantly influences the CSH, which is one of the factors responsible for bacterial adhesion to biotic and abiotic surfaces (23). Hence, the inhibitory potential of citral on EPS production could possibly affect the CSH of A. baumannii. To check this, the effect of citral on the CSH of A. baumannii was assessed, and the data revealed that citral significantly affected the CSH of A. baumannii (Fig. 4).

The strong antibiofilm potential of citral against A. baumannii prompted us to elucidate the underlying molecular mechanism of action by proteomic analysis. Therefore, the cellular proteomes of control and citral (200-μg/ml)-treated A. baumannii were assessed by 2DGE (Fig. 6), and differentially regulated protein spots were identified using MALDI-TOF/TOF analysis (Tables 1 and 2). To validate the proteomic results and evaluate the influence of citral on biofilm- and virulence factor-associated genes, qPCR analysis was performed (Fig. 10). The results unveiled the downregulation of the gene expression of the response regulator of the biofilm-associated TCS (*bfmR*), the biofilm-associated protein (*bap*), the CsuA/BABCDE chaperone-usher complex (*csuA-csuB*), the outer membrane protein (*ompA*), the PNAG-encoding locus (*pgaA* and *pgaC*), catalase (*katE*), and superoxide dismutase (*sodB*), and they were well correlated with the results of the proteomic analysis.

Furthermore, *in silico* analyses were performed to evaluate the interaction and functional attributes of differentially expressed proteins of A. baumannii. PPI and functional enrichment analyses revealed that upregulated proteins are predicted to be involved in diverse cellular, metabolic, and oxidation reduction processes such as biosynthetic and nitrogen compound processes (Fig. 9A). The molecular functions of these proteins were correlated with several binding, catalytic, ligase, and oxidoreductase activities (Fig. 9B). In cellular components, upregulated proteins were present in the protein-containing complex, membrane, cytoplasm, and cell periphery (Fig. 9C). Downregulated proteins were involved in the tricarboxylic acid (TCA) cycle, cellular oxidant detoxification, the response to oxidative stress, reactive oxygen species metabolic processes, protein refolding, and diverse catabolic processes (Fig. 9D). In the molecular functional analysis, these proteins were found to be involved in transferase, catalytic, carbon-carbon lyase, antioxidant, efflux transmembrane transporter, and various binding activities (Fig. 9E). These downregulated proteins are present in the cell wall, plasma membrane, cell envelope, cell outer membrane, protein-containing complex, external encapsulating structure, and type II protein secretion system complex (Fig. 9F). PPI and functional enrichment analyses revealed the diverse mechanisms of differentially regulated proteins of A. baumannii, indicating that citral has multitarget efficacy.

Additionally, C-G-N analysis was done to assess the interaction of downregulated protein-coding and transcriptional genes of A. baumannii with virulence and essential genes retrieved from the VFDB and DEG. C-G-N analysis unveiled the (VFDB, MALDI-TOF/TOF, and qPCR) interactions between genes such as *ompA*, *bfmR*, *adeH*, *adeF*, *adeG*, *plcD*, *plc*, *bap*, *csuA-csuB*, *pgaC*, *pgaA*, *sodB*, and *katE* (Fig. 11A and B).

The *ompA* and *bfmR* genes play important roles in bacterial adherence and the two-component system of A. baumannii. *adeH*, *adeF*, *adeG*, *plcD*, and *plc* are associated with the efflux pump, autoinducer, transport, and lipase enzyme production. The *bap*, *csuA-csuB*, *pgaC*, and *pgaA* genes are involved in PNAG synthesis and fimbria-associated motility. *sodB* and *katE* are responsible for antioxidant activity and quorum-sensing-mediated biofilm formation in A. baumannii (24, 25). These results showed that the bioactive molecule citral hindered biofilm formation by altering various virulence mechanisms of A. baumannii. Overall, systematic network interactions and Venn intersections revealed the plausible mode of action and multitarget efficacy of citral. Furthermore, Venn and network analyses showed no interactions between the DEG and downregulated genes (Fig. 11C and D), and they suggest that citral does not affect the normal growth and metabolism of A. baumannii.

The results of the proteomic analysis showed that citral treatment extremely affected the expression of chaperonins in A. baumannii. Chaperonins play a key role in protein folding, the heat shock response, and cellular homeostasis in bacteria. In addition, aminoglycoside antibiotics affect bacteria by inducing protein misfolding. Bacterial chaperonins encounter protein misfolding caused by aminoglycoside antibiotics and thereby promote bacterial survival against aminoglycoside antibiotics (26). Cardoso et al. reported that the overexpression of chaperonin enhanced the resistance of A. baumannii to aminoglycoside antibiotics (27). In order to validate the downregulation of chaperonins by citral, the sensitivity of A. baumannii to antibiotics was assessed, and the results showed that citral treatment increases the susceptibility of A. baumannii to conventional antibiotics (Fig. 12). Furthermore, universal stress protein A (UspA) and antioxidant enzymes such as SOD and catalase (Kat) of A. baumannii were downregulated in the citral-treated sample. UspA is involved in the mechanisms of physiological stress resistance in A. baumannii. On the other hand, UspA is important for the protection of A. baumannii from H_2_O_2_ (28). Therefore, the effect of citral treatment on the sensitivity of A. baumannii to H_2_O_2_ was assessed, and the results showed that citral treatment enhanced the sensitivity of A. baumannii to H_2_O_2_ (Fig. 12C). Antioxidant enzymes (Sod, Kat, and alkyl hydroperoxide reductase [Ahp]) are involved in the process of detoxification of ROS generated during bacterial metabolism. In bacteria, superoxide is produced through the electron transport chain, bacterial exposure to the host immune system, and antibiotics. Superoxide is then converted into H_2_O_2_ by Sod and neutralized into 2H_2_O and O_2_ by Kat and Ahp, respectively. In A. baumannii, the production of Kat and Sod comes under quorum-sensing-mediated biofilm formation (29, 30).

Proteomic analysis revealed that citral treatment affects the expression of Sod, Kat, and Ahp, which was further validated by native PAGE analysis (Fig. 13A and B). The mutation in SOD significantly affected the surface motility of A. baumannii (31), and this result goes well with the results of the motility assay in the present study (Fig. 14). The response regulator receiver domain protein (AdeRS) is a two-component system of A. baumannii that regulates the expression of the efflux pump (RND family), TetR family transcriptional regulators, and LysR family transcriptional regulators. Thereby, A. baumannii acquires efflux pump-mediated antibiotic resistance, bacterial motility, and biofilm formation (32–35). AdeRS and its associated proteins such as efflux pump (RND family), TetR family transcriptional regulator, and LysR family transcriptional regulator proteins were found to be downregulated by citral, which is well correlated with the results of biofilm assays and different types of motility of A. baumannii (Fig. 13). Citral treatment affected the secretion systems of A. baumannii, such as the type II and type VI secretion systems (LysM domain protein). The type II secretion system of A. baumannii is involved in the process of virulence enzyme production, including lipase and protease. Hence, it was validated by lipolytic quantification and protease assays, and the results revealed reductions in lipase and protease production in A. baumannii treated with citral (Fig. 13D). The activation of the type II secretion system is dependent on the expression of chaperones. On the other hand, the expression of chaperonin was also suppressed by citral treatment. The type VI secretion system is an important virulence factor of A. baumannii that promotes the injection of toxin proteins into host organisms and other competitive bacteria (36).

Proteomic analysis revealed that citral treatment affected iron acquisition and iron homeostasis proteins such as bacterioferritin, the ring-hydroxylating beta subunit, and the TonB-dependent siderophore receptor protein. Iron is an essential element for bacterial cellular processes and pathogenesis. Bacterioferritin and ring-hydroxylating beta proteins are involved in the iron acquisition process and thereby help bacterial growth and pathogenesis in host organisms (37, 38). During pathogenesis, the host organism generates free Fe^3+^ molecules, and the accumulation of Fe^3+^ causes oxidative stress to bacteria. The iron-chelating protein siderophore and TonB-dependent siderophore receptor protein-mediated iron-chelating process is involved in the protection of bacteria from oxidative stress (39, 40). Due to the negative effect of citral on the iron acquisition mechanism of A. baumannii, the sensitivity of A. baumannii to H_2_O_2_ was found to be enhanced (Fig. 12C). In addition, hemolysin is a notable virulence factor produced by bacteria to release iron from the hemoglobin of red blood cells for bacterial growth and development. In order to assess the effect of citral on hemolysin production in A. baumannii, a hemolysin assay was performed, and the results revealed a marked reduction of hemolysin in A. baumannii cells treated with citral (Fig. 13D) (41). These results suggest that citral strongly affects the iron acquisition and iron homeostasis of A. baumannii. The RstA (BfmR) transcriptional regulatory protein of A. baumannii has been found to be downregulated by citral treatment. BfmR is a biofilm-associated master regulatory TCS, and it regulates the biofilm formation, bacterial adherence, and chaperone-usher assembly system (CsuA/BABCDE) of A. baumannii. The chaperone-usher assembly system controls A. baumannii motility; therefore, the effect of citral on the motility of A. baumannii was checked, and the motility of A. baumannii was found to be affected by citral treatment (Fig. 14). Additionally, BfmR supports the survival of A. baumannii in human serum and was also assessed by a blood survival assay, and the results showed that citral treatment enhanced the sensitivity of A. baumannii to the host immune system compared to the control sample (Fig. 12C) (42–44). The H-NS histone family protein regulates various functions of bacteria, such as the expression of outer membrane proteins, motility, biofilm formation, and extracellular polysaccharide synthesis (45, 46). Citral treatment affected the H-NS histone family protein, which could be one of the reasons for the inhibitory effect of citral on the biofilm, EPS, and motility of A. baumannii. Citral treatment downregulated proteins that play an essential role in the TCA cycle, such as isocitrate lyase, isocitrate dehydrogenase, fumarate hydratase class II, malate synthase G, malate dehydrogenase, fructose-bisphosphate aldolase, and a glyoxalase family protein. Previous studies have shown that the overexpression of TCA cycle-associated proteins increased the biofilm formation, virulence factor production, and antibiotic resistance of A. baumannii (47, 48). Our results indicate that citral treatment downregulated TCA cycle-associated proteins, thereby controlling biofilm formation and virulence factors of A. baumannii.

**FIG 16:**
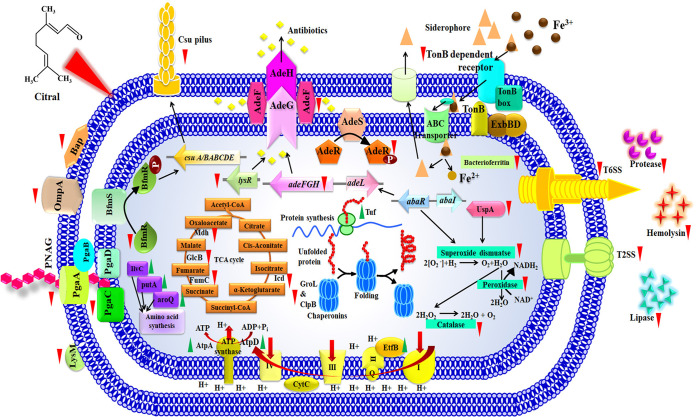
Multitarget efficacy of citral. The schematic diagram shows the proteins and pathways of A. baumannii targeted by citral. T6SS, type VI secretion system.

In this study, proteomic analysis revealed that citral treatment upregulated proteins associated with protein synthesis, amino acid biosynthesis, ATP synthesis, the electron transport chain, fatty acid biosynthesis, and nucleic acid synthesis (Table 2). These are biologically important processes for the survival of bacteria. Hence, the analysis confirmed that citral at a 200-μg/ml concentration does not affect the proteins required for the survival of A. baumannii, which is well correlated with the results of growth curve and Alamar Blue assays. Therefore, the chance of A. baumannii gaining resistance to citral can be very small or nil. In addition, a glycosyl hydrolase family protein was found to be upregulated upon treatment with citral, and it is involved in the disassembly of existing biofilms (49). Hence, the upregulation of the glycosyl hydrolase family protein could increase biofilm disruption in citral-treated samples. Finally, cytotoxic analyses revealed the nonfatal effect of citral on PBMCs (Fig. 15). Overall, this holistic study unveils the multitarget efficacy of citral to impede the biofilm formation and virulence of A. baumannii. Various virulence regulatory systems targeted by citral are schematically presented in Fig. 15. Citral treatment majorly impacted the two-component system BfmRS, which regulates biofilm formation, motility, EPS synthesis, iron transport, toxin secretory systems, the AdeABC system that provides antibiotic resistance, and the antioxidant system. Interestingly, proteins involved in electron transport, amino acid synthesis, and protein synthesis are positively regulated by citral. The present study suggests that citral could be safe and can be considered for further therapeutic investigations for the treatment of biofilm-associated infections by A. baumannii (Fig. 16).

### Conclusion.

In conclusion, the present study revealed the antibiofilm potential of citral without causing any harmful effect on the growth and metabolism of A. baumannii. The global proteomics-based analysis unveiled that citral treatment affected biofilm formation, antibiotic resistance, iron homeostasis, the antioxidant defense system, the iron acquisition system, and the type II and type IV secretion systems of A. baumannii. In addition, citral treatment positively regulated the proteins associated with the growth and metabolism of A. baumannii, which suggests that the possibility of drug resistance to citral is very small. Furthermore, citral exhibited nontoxic effects on human PBMCs, and hence, citral could be a safe therapeutic compound for the treatment of infections caused by A. baumannii. Altogether, the results of the present study emphasize the multitarget potential of citral to inhibit the biofilm and virulence factors of A. baumannii.

## MATERIALS AND METHODS

### Ethics statement.

In the current study, healthy human blood was used for blood survival and cytotoxicity analyses, and sheep blood was used for hemolysis assays. The human blood sample was collected from a healthy individual by a technically skilled person, and written informed consent was obtained. The use of the human blood sample and the experimental protocol were assessed and approved by the Institutional Ethics Committee (IEC), Alagappa University, Karaikudi (IEC reference no. IEC/AU/2018/4). The sheep blood was collected from the municipality slaughterhouse, Karaikudi, Tamil Nadu, India. As sheep blood is discarded in the slaughterhouse, specific ethical permission is not required.

### Bacterial strain and growth conditions.

A. baumannii MTCC 9829 was obtained from the Microbial Type Culture Collection (MTCC), India. It was grown in tryptone soya broth (Hi-Media, India), incubated at 37°C for 24 h, and stored at 4°C. A single isolated colony of A. baumannii was inoculated into tryptone soya broth (TSB) supplemented with 1% sucrose and 0.5% yeast extract (TSBSY), incubated at 37°C overnight, adjusted to 10^6^ cells/ml, and used for all the experiments.

### Preparation of phytocompound stock solutions.

Citral (Alfa Aesar, India), chlorogenic acid (Sigma-Aldrich, India), vanillic acid (Alfa Aesar, India), ferulic acid (Sigma-Aldrich, India), gallic acid (Sigma-Aldrich, India), geraniol (Sigma-Aldrich, India), catechin, and naringin hydrate (Sigma-Aldrich, India) were dissolved in methanol. Sterile MilliQ water was used for dissolving caffeine and l-glutathione (Alfa Aesar, India). All the phytocompounds were prepared as 10-mg/ml stock solutions and stored at 4°C for future use.

### Evaluation of antibiofilm activities of phytocompounds.

The phytocompound(s) (250 μg/ml) was added to 200 μl of TSBSY containing 1% of a culture of A. baumannii grown overnight in a 96-well microtiter plate and incubated at 37°C for 24 h. TSBSY alone and TSBSY inoculated with 1% of the culture grown overnight (adjusted to 10^6^ cells/ml) were considered the blank and the control, respectively. After incubation, the plate was read at 600 nm to assess the optical density (OD) of the culture, and planktonic cells were discarded. Next, the wells were washed thoroughly with sterile distilled water. A crystal violet (0.4%) solution was used to stain the wells for 10 min, and the wells were washed with sterile distilled water followed by air drying. The wells were destained using 30% glacial acetic acid for 10 min, and the absorbance was read at 570 nm. Finally, the antibiofilm activity of phytocompounds and their percentage of inhibition were measured by using the following formula: biofilm inhibition (%) = [(control OD_570_ − treated OD_570_)/control OD_570_] × 100 (50).

### Determination of the MIC and MBIC of citral.

Citral, which showed antibiofilm activity against A. baumannii, was added at increasing concentrations (25 to 400 μg/ml) to wells containing 1 ml of TSBSY in a sterile 24-well microtiter plate and inoculated with 1% of a culture of A. baumannii grown overnight. The plate was incubated at 37°C for 24 h. After incubation, the OD of culture was obtained, and biofilm quantification was performed as described above. The MIC and minimal biofilm-inhibitory concentration (MBIC) were fixed as the maximum growth inhibition and maximum biofilm reduction at the lowest concentration of citral, respectively (51).

### Growth curve analysis.

To determine the effect of citral on the growth of A. baumannii, citral (200 μg/ml) was added to 100 ml of TSBSY containing 1% of a culture of A. baumannii grown overnight. TSBSY inoculated with 1% of the A. baumannii culture with the addition of 1 ml of methanol was considered the vehicle control, and TSBSY with citral added and without an inoculum was considered the blank. Next, the flasks were incubated at 37°C, and the ODs of control and treated samples were measured at 600 nm for 0 to 24 h at serial intervals of 1 h (51).

### Alamar Blue assay.

An Alamar Blue assay was performed to quantitatively analyze the influence of citral on the cell viability of A. baumannii. Briefly, a culture of A. baumannii grown overnight was used to inoculate 1 ml of TSBSY without and with citral at 200 μg/ml and allowed to grow for 24 h at 37°C. The control and treated cells were collected by centrifugation at 8,000 rpm for 10 min, washed twice with phosphate-buffered saline (PBS), and resuspended in PBS. The Alamar Blue (0.1-ml) substrate (1 mg/ml in PBS) was mixed with 0.8 ml of control and treated cell suspensions, and the samples were incubated in the dark at 37°C for 4 h. After incubation, the samples were centrifuged at 8,000 rpm for 10 min. Next, the fluorescence intensity of the supernatants was quantified spectrophotometrically with an excitation wavelength of 560 nm and an emission wavelength of 590 nm. Cell viability is directly proportional to the reduction of the level of blue color to pink (51).

### Microscopic analyses of A. baumannii biofilms.

The biofilm architecture of control and citral-treated samples was qualitatively analyzed by microscopic techniques. A biofilm assay was performed on 1- by 1-cm glass slides in a 24-well plate containing 1 ml of TSBSY and 1% of the inoculum, without and with 200 μg/ml of citral. The assay plate was incubated at 37°C for 24 h. After incubation, the slides were stained and observed using microscopes (52).

### (i) Light microscopic analysis.

For light microscopic analysis, the slides were washed gently with sterile PBS, stained with 0.4% crystal violet for 1 min, washed again with sterile PBS to remove the excess stain, and air dried. The air-dried glass slides were examined under a light microscope (Eclipse Ti-S; Nikon, Tokyo, Japan) at a ×400 magnification.

### (ii) Confocal laser scanning microscopy analysis.

For confocal laser scanning microscopy (CLSM), the slides were washed with sterile PBS, stained using 0.1% acridine orange under dark conditions for 5 min, washed with sterile PBS to remove the unbound stain, and air dried. Next, the glass slides were observed by CLSM (Zeiss LSM-710; Carl Zeiss, Oberkochen, Germany) at a magnification of ×200.

### (iii) Scanning electron microscopy analysis.

For scanning electron microscopy (SEM) analysis, the glass slides were washed with sterile PBS, fixed with 2% glutaraldehyde for 8 h at 4°C, and washed again with sterile PBS. Next, the slides were dehydrated with increasing concentrations of ethanol (20, 40, 60, 80, and 100%). After gold sputtering, the slides were observed by SEM (Vega 3; Tescan, Czech Republic).

### MATH assay.

The effect of citral on the cell surface hydrophobicity (CSH) of A. baumannii was determined by a microbial adherence to hydrocarbon (MATH) assay (50). The cells of control and citral-treated cultures were harvested by centrifugation at 8,000 rpm for 10 min and resuspended in sterile PBS. An equal volume of toluene was added to 1 ml of the cell suspension (OD of 0.8 ± 0.05), and the mixture was vortexed for 2 min. After vortexing, the tubes were kept under undisturbed conditions for 6 h for the separation of the aqueous phase and toluene phase. The toluene phase was discarded, and the remaining solvent traces were completely eliminated by keeping the tubes open at 37°C overnight. The OD of the aqueous phase was read at 600 nm, and the CSH was measured as the hydrophobicity index, using the following formula: hydrophobicity index = [1 − (OD_600_ after vortexing/OD_600_ before vortexing)] × 100.

### Quantification of EPS.

EPS quantification was done to compare the total amounts of EPS production in control and treated samples. Initially, 100 ml of A. baumannii cultures grown in the absence and presence of citral (200 μg/ml) at 37°C for 24 h were centrifuged at 8,000 rpm for 10 min in order to separate cell pellets and the cell-free culture supernatant (CFCS). An equal volume of isotonic buffer (10 mM Tris-HCl [pH 8.0], 10 mM EDTA, 2.5% NaCl) was added to the cell pellet, and the mixture was incubated at 4°C overnight to isolate cell-bound EPS. After incubation overnight, the cell suspension was centrifuged at 8,000 rpm for 10 min, and the supernatant of the cell suspension was mixed with the CFCS. Next, ice-cold ethanol and the CFCS containing cell-bound EPS were mixed at a ratio of 3:1 and incubated at −20°C overnight. The EPS mixture was then centrifuged at 12,000 rpm at 4°C for 30 min in order to separate the pellet. The pellet was then suspended in 70% ethanol and dried in a vacuum evaporator. Finally, the dried EPS was weighed, and EPS inhibition was calculated using the following formula: EPS (%) = (control weight − treated weight/control weight) × 100 (50).

### Cellular protein extraction and sodium dodecyl sulfate-polyacrylamide gel electrophoresis analysis.

Initially, A. baumannii was allowed to grow in the absence and presence of citral (200 μg/ml) in 100 ml of TSBSY for 24 h at 37°C with constant shaking at 160 rpm, in biological triplicates. After incubation, the cell pellet was collected by centrifugation at 8,000 rpm for 15 min at 4°C. Next, the pellet was washed three times with PBS to remove debris and resuspended in 2 ml of 20 mM Tris-HCl containing 1% protease inhibitor cocktail. The samples were then subjected to sonication on ice using an ultrasonicator (Sonics VCX 750) with a 30% amplitude and a pulse time of 10-s on-and-off cycles for 10 min. After sonication, samples were centrifuged at 12,000 rpm for 30 min at 4°C, and the supernatant containing proteins was cleaned using the 2-D Clean-Up kit (GE Healthcare) according to the manufacturer’s instructions. Next, purified protein samples were dissolved in sample buffer {7 M urea, 2 M thiourea, 40 mM dithiothreitol (DTT), and 4% 3-[(3-cholamidopropyl)-dimethylammonio]-1-propanesulfonate (CHAPS)} and quantified by the Bradford method using a Bio-Rad protein assay kit. A total of 50 μg of protein from the control/citral-treated sample was loaded onto a 12% sodium dodecyl sulfate-polyacrylamide gel electrophoresis (SDS-PAGE) gel (SE 600 vertical unit; GE Healthcare) and run at 50 V for 30 min for the stacking gel and at 100 V for 5 h for the separating gel. After running SDS-PAGE, the gels were soaked in a fixative solution (10% glacial acetic acid, 40% methanol, and 50% water) for 6 h and washed three times with distilled water at 20-min intervals. Colloidal Coomassie brilliant blue (CBB) G-250 staining solution (10% orthophosphoric acid, 10% ammonium sulfate, 20% methanol, and 0.12% CBB) was used to stain the gels for 12 h with gentle agitation. After staining, the CBB solution was removed, and gels were destained with distilled water. Next, the gels were imaged using an Image Scanner III system (GE Healthcare) equipped with LabScan 6.0 software (52).

### Isoelectric focusing and two-dimensional gel electrophoresis.

Prior to isoelectric focusing (IEF), six immobilized pH gradient (IPG) strips (Immobiline DryStrip pH 4 to 7, 18 cm) were rehydrated for 12 h at 20°C by adding 350 μl (for each strip) of rehydration buffer (7 M urea, 2 M thiourea, 2% CHAPS, 0.5% IPG buffer [pH 4 to 7], 0.002% bromophenol blue, and 12.5 mg/ml Destreak reagent [GE Healthcare]) containing 500 μg of protein extracted from the control/citral-treated sample per strip (biological triplicates). After rehydration, IPG strips were placed on mineral oil in an IEF system (Ettan IPGphor 3; GE Healthcare), and IEF was carried out by applying a 75-μA current per IPG strip under the following conditions: 100 V (2-h step), 100 to 500 V (3-h gradient), 500 V (2-h step), 500 to 5,000 V (3-h gradient), 5,000 V (2-h step), 5,000 to 8,000 V (3-h gradient), 8,000 V (2-h step), 8,000 to 10,000 V (3-h gradient), and 10,000 V (2-h step) at 20°C for 22 h. Prior to two-dimensional gel electrophoresis (2DGE), the IPG strips were equilibrated with two different equilibration buffers, equilibration buffer I (6 M urea, 30% glycerol, 2% SDS, 0.002% bromophenol blue, and 1% DTT in 50 mM Tris-HCl buffer [pH 8.8]) for 15 min and equilibration buffer II (6 M urea, 30% glycerol, 2% SDS, 0.002% bromophenol blue, and 2.5% iodoacetamide [IAA] in 50 mM Tris-HCl buffer [pH 8.8]) for 15 min. Next, equilibrated IPG strips were placed on top of 12% gels and sealed with a 0.3% agarose solution. The Ettan DALTsix electrophoresis system (GE Healthcare) was used for electrophoresis under the following program conditions: 100 V (200 mA) for 1 h and 150 V (300 mA) for 8 h. After electrophoresis, the gels were fixed, stained by CBB according to the above-described method, and imaged at a 300-dpi (dots per inch) resolution using an Image Scanner III system (GE Healthcare) equipped with LabScan 6.0 software (53).

### Identification of differentially expressed proteins by image analysis and in-gel digestion of proteins.

ImageMaster 2D Platinum software (GE Healthcare) was used according to the manufacturer’s instructions to assess the differentially expressed protein spots in control and citral-treated gels. After image analysis, differentiated protein spots of >1.5-fold were excised using sterile cut tips. Next, the gel pieces were washed three times with a destaining solution (25 mM ammonium bicarbonate in 50% mass spectrometry [MS]-grade water and 50% acetonitrile [ACN]), dehydrated with MS-grade ACN for 10 min, and vacuum dried for 30 min. The gel pieces were taken for reduction (25 mM ACN and 10 mM DTT in MS-grade water) for 30 min at 55°C and alkylation (25 mM ACN and 55 mM IAA in MS-grade water) at 37°C for 30 min. Again, gel pieces were dehydrated with ACN and dried under a vacuum. Next, the gel pieces were digested with a trypsin solution (5 μl) containing 10 mM ammonium bicarbonate and 400 ng of trypsin in 10% ACN, covered with overlay buffer (25 μl) containing 40 mM ammonium bicarbonate in 10% ACN, and incubated at 37°C in a water bath for 16 h. After trypsin digestion, peptides were eluted from gel pieces by adding 25 μl of 60% ACN containing 0.1% trifluoroacetic acid (TFA) and sonicated for 5 min. After sonication, tubes were centrifuged at 12,000 rpm for 10 min, and the supernatant containing peptides was dried under a vacuum for 90 min (53). Next, the eluted peptides were reconstituted in a peptide resuspension solution (0.1% TFA in 5% ACN). To purify the peptides, C_18_ Ziptips (Millipore, Merck) were used according to the manufacturer’s protocol.

### MALDI-TOF/TOF mass spectrometry analysis.

Prior to mass spectrometry analysis, the matrix-assisted laser desorption ionization–time of flight/time of flight (MALDI-TOF/TOF) mass spectrometer (Axima Performance; Shimadzu Biotech) was calibrated using TOF-Mix (LaserBio Labs, France) containing a mixture of seven peptides. After calibration, 1 μl of a matrix solution (10 mg of an alpha-cyano-4- hydroxycinnamic acid matrix and 0.1% formic acid in 1 ml of 60% methanol) was mixed with 1 μl of the peptide sample and spotted onto the target plate. Next, mass spectrometry analysis was performed, and peptide mass spectra were obtained by MALDI-TOF/TOF analysis using Shimadzu launch pad-MALDI MS software. Monoisotopic peaks were selected between 700 and 4,000 Da with MALDI MS software and analyzed using the MS-Fit (http://prospector.ucsf.edu) online database with the following parameters: trypsin enzyme digestion with two missed cleavages per peptide, 1.5-Da mass tolerances with cysteine carbamidomethylation and methionine oxidation, acetylation of the N terminus, and phosphorylation of S, T, and Y amino acids (16, 19).

### Quantitative real-time PCR analysis.

To assess the influence of citral on biofilm-associated genes such as *bfmR*, *bap*, *csuA-csuB*, *ompA*, *pgaA*, *pgaC*, *katE*, and *sodB*, quantitative real-time PCR (qPCR) analysis was performed. Initially, total RNA was isolated from control and citral-treated A. baumannii cells by the TRIzol method, and cDNA conversion was done using a high-capacity cDNA reverse transcription kit (Applied Biosystems). Next, qPCR was performed on a 7500 thermal cycler sequence detection system (Applied Biosystems Inc., Foster City, CA, USA) using Power SYBR green PCR master mix (Applied Biosystems) according to the manufacturer’s instructions. The gene expression level was calculated after normalizing the cycle threshold (*C_T_*) values of biofilm-associated genes with a housekeeping gene (*rplB*) using the 2^−ΔΔ^*^CT^* method. Details of the primer sequences for biofilm-associated genes are listed in Table 3 (16, 24).

**TABLE 3:** Primers used for qPCR analysis

Gene	Forward primer	Reverse primer
*bfmR*	5′-CTGGTAGGTAATGCAGTTCG-3′	5′-GAGAGACCCAAACCATAACC-3′
*bap*	5′-GTACTCCAGCAACGGTTGTA-3′	5′-GAAGGATCTGCTGTATTCCA-3′
*csuA-csuB*	5′-ATGCGGTAAATACTCAAGCA-3′	5′-TCACAGAAATATTGCCACCT-3′
*ompA*	5′-CTCTTGCTGGCTTAAACGTA-3′	5′-GCAATTTCTGGCTTGTATTG-3′
*pgaA*	5′-CACATGGCAAAAAGATGAAT-3′	5′-CGTAGAAACCTCGAACAGTG-3′
*pgaC*	5′-CAGTGGTATGGCGTGATATT-3′	5′-GGTACTGCAACAACACTGGT-3′
*katE*	5′-GTGTCCGGTTCAGGTTTTAC-3’	5′-GGATTCTTGACAGACCCAAC-3′
*sodB*	5′-TGAACCGAATTGAGTTGTTG-3′	5′-TTCAACAATGCTGCTCAAGT-3′
*rplB*	5′-GGTCGTAATAACAACGGTCA-3′	5′-AATAATGCAATATGCGCTGT-3′

### Signaling network and gene enrichment analysis.

Protein-protein interactions (PPIs) of differentially expressed proteins from A. baumannii were imputed using STRING v11.0 (https://string-db.org/) with a high-confidence score of 0.7 (54). Interactions of these proteins and their details were exported in TSV (tab-separated value) file format from the STRING tool, and the same was imported to ClueGO v2.5.7/CluePedia v1.5.7 for gene ontology (GO) analysis. GO enrichment was performed using the ClueGO/CluePedia plug-in of Cytoscape v3.8.0 based on the two-sided hypergeometric test (statistical test) and Bonferroni step-down (correction method) with a kappa score threshold level of 0.38 (55–57).

### Comparative gene network analysis.

Comprehensive information on virulence and essential genes of A. baumannii was collected from the Virulence Factor Database (VFDB) (25) and the Database of Essential Genes (DEG) (58), respectively. The collected information and downregulated protein-coding genes from MALDI-TOF/TOF and qPCR analyses were compared using Draw Venn Diagram (http://bioinformatics.psb.ugent.be/webtools/Venn/), and the molecular interaction network was visualized using Cytoscape v3.8.0.

### Antibiotic sensitivity assay.

The test tubes were supplemented with 2 ml of TSBSY and various concentrations of antibiotics (amikacin, cefotaxime, ciprofloxacin, and gentamicin) alone and with citral at 200 μg/ml. For inoculation, 1% of a culture of A. baumannii grown overnight was added, and the mixture was incubated at 37°C for 24 h. After incubation, the culture was read at 600 nm, and the percentage of inhibition was measured using the following formula: percent inhibition = (control OD_600_ − treated OD_600_/control OD_600_) × 100 (50).

### Qualitative analysis of catalase and superoxide dismutase activities by native PAGE.

Control and citral (200-μg/ml)-treated A. baumannii cells were pelleted by centrifugation at 8,000 rpm for 10 min at 4°C and suspended in ice-cold 50 mM potassium phosphate (KP_i_) buffer (pH 7). Intracellular protein was isolated by sonication on ice. After sonication, cell debris was removed by centrifugation at 8,000 rpm for 20 min, and protein was quantified by the Bradford method. A total of 50 μg of protein from control/citral-treated samples was loaded on two different nondenaturing polyacrylamide gels at 8% for catalase activity and 12% for superoxide dismutase (SOD) activity, respectively. Electrophoresis was carried out at 50 V at 20°C using running buffer containing 50 mM Tris, 300 mM glycine, and 1.8 mM EDTA for 6 h (52, 53).

### (i) Catalase activity.

For catalase activity, after electrophoresis, the gel (8%) was washed three times with 50 mM KP_i_ buffer. The gel was then incubated with a 4 mM H_2_O_2_ solution for 10 min and stained with a solution containing 1% potassium ferricyanide and 1% ferric chloride. The gel was allowed to stain until the appearance of a dark blue-green background with clear bands and further washed with distilled water.

### (ii) SOD activity.

For SOD activity, the 12% gel was incubated in 100 ml of freshly prepared 50 mM KP_i_ buffer with substrates such as 0.1 mM EDTA, 2 mg riboflavin, and 16 mg nitroblue tetrazolium (NBT). Next, tetramethylethylenediamine (TEMED) (400 μl) was added to the substrate solution, and the mixture was incubated at 37°C in the dark for 1 h to reduce NBT. After incubation, the gel was washed, immersed in 50 mM KP_i_, and exposed to light. The SOD activity was assessed by the appearance of achromatic bands in the gel with a purple background, and the gel was immediately imaged.

### H_2_O_2_ susceptibility assay.

A. baumannii was grown for 24 h in TSBSY without and with citral at 200 mg/ml. Next, cells were collected by centrifugation at 8,000 rpm for 10 min and resuspended in PBS containing 1 mM H_2_O_2_. The samples were incubated at 37°C for 1 h with shaking at 160 rpm, and viable cells were enumerated by the CFU method (51).

### Blood survival assay.

Citral-treated and untreated A. baumannii cells were suspended in sterile PBS. The cell suspension was added to healthy human blood at a ratio of 1:4 and mixed thoroughly. The mixtures were incubated in a shaking incubator at 37°C for 3 h. The viable cells in control and treated samples were quantified by the serial dilution method (59).

### Lipolysis assay.

The extracellular lipase production of control and citral-treated A. baumannii was assessed using *p*-nitrophenyl palmitate (pNPP) as the substrate. Briefly, A. baumannii was grown in the absence and presence of citral (200 μg/ml) for 24 h at 37°C. After incubation, the CFCS was collected by centrifugation at 8,000 rpm for 10 min. The pNPP substrate was prepared by mixing 1 volume of pNPP (0.3% in 2-propanol) with 9 volumes of 50 mM Na_2_PO_4_ buffer (pH 8.0) containing sodium deoxycholate (0.2%) and gummi arabicum (0.1%). Next, 100 μl of the CFCS was added to 900 μl of the pNPP substrate, incubated for 1 h at 37°C in the dark, and then centrifuged at 12,000 rpm for 5 min; the absorbance of the supernatant was measured at 410 nm; and the percentage of inhibition was calculated according to a previously described formula (9).

### Protease assay.

The proteolytic activity of A. baumannii control and citral-treated samples was analyzed by mixing 100 ml of a protease substrate solution (0.2% azocasein dissolved in 0.05 M Tris-hydrochloride) with 100 ml of the CFCS of A. baumannii samples, and the mixture was incubated at 37°C for 30 min. To terminate the reactions, 10% trichloroacetic acid (500 μl) was added to the mixture, and the mixture was incubated at −20°C for 15 min and centrifuged at 8,000 rpm for 15 min. Next, the supernatant absorbance was measured at 400 nm, and the percentage of inhibition was calculated (9).

### Hemolysin assay.

To analyze the effect of citral on hemolysin production in A. baumannii, the CFCS was collected from A. baumannii grown in the absence and presence of citral for 24 h at 37°C. Next, 100 μl of the CFCS of the control/citral-treated sample was mixed with 900 μl of 2% sheep red blood cells in PBS and incubated at 37°C for 1 h. Subsequently, the samples were centrifuged at 3,000 rpm for 15 min, the absorbance of the supernatant was read at 530 nm, and the percentage of inhibition was calculated (9).

### Motility assays.

Motility assays were performed to assess the influence of the MBIC (200 μg/ml) of citral on the motility of A. baumannii (60, 61).

**(i) Swimming.** TSB (0.3% agar) plates were prepared with 200 μg/ml of citral, and plates with the corresponding volume of methanol were used as controls. A 5-μl culture was spotted onto the middle region of the plate and incubated at 37°C for 72 h. After incubation, the swimming motility of control and treated cells was measured.

**(ii) Swarming.** A 10-μl A. baumannii culture was spotted onto the middle region of the TSB plate (0.5% agar) supplemented without and with citral (200 μg/ml) and incubated at 37°C for 72 h. After incubation, the zone of swarming motility of control and treated cells was observed.

**(iii) Twitching.** A. baumannii cells were stab inoculated using a toothpick into the bottom of 1% agar-containing tryptic soya agar (TSA) in 6-well polystyrene plates without and with citral (200 μg/ml). The plate was incubated for 72 h at 37°C. The A. baumannii twitching mobility on the polystyrene surface was examined by removing the agar, washing with sterile PBS, and staining with a 0.3% crystal violet solution.

### Cytotoxicity assay.

Peripheral blood mononuclear cells (PBMCs) were isolated from freshly collected blood by gently adding it to a tube containing lymphocyte separation medium (Histopaque; Sigma-Aldrich, India). Next, the tube was centrifuged at 2,500 rpm at 20°C for 30 min, and the buffy coat layer was collected and washed with RPMI 1640 medium. Next, PBMCs were suspended in complete medium (1% Anti-Anti [antibiotic-antimycotic] solution and 10% fetal bovine serum in RPMI 1640 medium) and adjusted to 1 × 10^5^ cells/ml. PBMCs were treated without and with citral (200 and 400 μg/ml) for 24 h at 37°C in a 5% CO_2_ incubator. A total of 1 mM H_2_O_2_ and 0.05% methanol were maintained as the positive control and the vehicle control, respectively. After incubation, cell viability was evaluated by an Alamar Blue assay and the trypan blue method (51).

### Statistical analysis.

All experiments were performed in three biological replicates with at least two technical replicates, and data are presented as means ± standard deviations (SD). Significant differences between the values of control and treated samples were analyzed by one-way analysis of variance (ANOVA) and Duncan’s *post hoc* test with a significant *P* value of ≤0.05 using SPSS statistical software package version 17.0 (SPSS, Chicago, IL, USA).
